# Diabetes-Related Ankyrin Repeat Protein (DARP/Ankrd23) Modifies Glucose Homeostasis by Modulating AMPK Activity in Skeletal Muscle

**DOI:** 10.1371/journal.pone.0138624

**Published:** 2015-09-23

**Authors:** Yoshiaki Shimoda, Kiyonari Matsuo, Youhei Kitamura, Kazunori Ono, Tomomi Ueyama, Satoaki Matoba, Hiroyuki Yamada, Tongbin Wu, Ju Chen, Noriaki Emoto, Koji Ikeda

**Affiliations:** 1 Department of Cardiology, Graduate School of Medical Science, Kyoto Prefectural University of Medicine, 465 Kajii, Kawaramachi-Hirokoji, Kamigyo, Kyoto 602–8566, Japan; 2 Department of Medicine, University of California San Diego, La Jolla, California, United States of America; 3 Department of Clinical Pharmacy, Kobe Pharmaceutical University, 4-19-1 Motoyama-Kitamachi, Higashinada, Kobe6588558, Japan; Universitat de Barcelona, SPAIN

## Abstract

Skeletal muscle is the major site for glucose disposal, the impairment of which closely associates with the glucose intolerance in diabetic patients. Diabetes-related ankyrin repeat protein (DARP/Ankrd23) is a member of muscle ankyrin repeat proteins, whose expression is enhanced in the skeletal muscle under diabetic conditions; however, its role in energy metabolism remains poorly understood. Here we report a novel role of DARP in the regulation of glucose homeostasis through modulating AMP-activated protein kinase (AMPK) activity. DARP is highly preferentially expressed in skeletal muscle, and its expression was substantially upregulated during myotube differentiation of C2C12 myoblasts. Interestingly, DARP-/- mice demonstrated better glucose tolerance despite similar body weight, while their insulin sensitivity did not differ from that in wildtype mice. We found that phosphorylation of AMPK, which mediates insulin-independent glucose uptake, in skeletal muscle was significantly enhanced in DARP-/- mice compared to that in wildtype mice. Gene silencing of DARP in C2C12 myotubes enhanced AMPK phosphorylation, whereas overexpression of DARP in C2C12 myoblasts reduced it. Moreover, DARP-silencing increased glucose uptake and oxidation in myotubes, which was abrogated by the treatment with AICAR, an AMPK activator. Of note, improved glucose tolerance in DARP-/- mice was abolished when mice were treated with AICAR. Mechanistically, gene silencing of DARP enhanced protein expression of LKB1 that is a major upstream kinase for AMPK in myotubes *in vitro* and the skeletal muscle *in vivo*. Together with the altered expression under diabetic conditions, our data strongly suggest that DARP plays an important role in the regulation of glucose homeostasis under physiological and pathological conditions, and thus DARP is a new therapeutic target for the treatment of diabetes mellitus.

## Introduction

Diabetes has been prevalence worldwide, and its morbidity is increasing rapidly, especially in developing countries [1]. Majority of diabetes is the type 2 diabetes that is closely associated with obesity. The primary cause of type 2 diabetes is insufficient insulin action in the body. Insulin targets various organs, but the principal insulin targets are liver, adipose tissue and skeletal muscle. Skeletal muscle accounts for ~75% of whole body insulin-mediated glucose uptake, and muscle insulin resistance could account for over 85% of the impairment in glucose disposal in type 2 diabetic subjects [2]. Therefore, improving muscle glucose uptake is a promising approach for the treatment of diabetic patients.

Glucose is transported into the cells through insulin-dependent and insulin-independent pathways. Upon insulin-stimulation, glucose transporter 4 (GLUT4) is translocated onto plasma membrane to mediate insulin-dependent glucose disposal into skeletal muscle and adipose tissue [3]. GLUT4 mediates insulin-independent glucose uptake as well, in response to exercise through AMP-activated protein kinase (AMPK) pathway [3]. AMPK is an energy-sensing enzyme that governs systemic energy metabolism, and is dysregulated in animals and humans with metabolic syndromes and/or type 2 diabetes [4,5]. On the other hand, GLUT1 is widely distributed in various tissues, and mediates constitutive, insulin-independent glucose uptake [6].

We previously identified a nuclear protein, termed diabetes-related ankyrin repeat protein (DARP; also known as CARP3 or ANKRD23), whose expression was differentially regulated in obese and/or insulin-resistant subjects [7]. DARP belongs to muscle ankyrin repeat protein (MARP) family that is considered to play a crucial role in the integration of cytoskeletal architecture, stress response and transcriptional regulation [8,9]. MARP family includes cardiac ankyrin repeat protein (CARP) that is predominantly expressed in the heart, and ankyrin repeat domain 2 (ANKRD2) that is widely expressed in many tissues with a high expression in the skeletal muscle and heart [8,9]. DARP is highly and preferentially expressed in skeletal muscle and its expression in skeletal muscle was enhanced in obese rats, while it was reduced during fasting in lean mice [7]. These observations suggest a possible role of DARP in energy metabolism, especially in skeletal muscle, but it remains largely unexplored. Here we investigated a potential role of DARP in glucose homeostasis using DARP-deficient (DARP-/-) mice, and found that loss of DARP enhanced systemic glucose tolerance without affecting insulin-sensitivity by increasing AMPK activity in skeletal muscle.

## Materials and Methods

### Materials

AICAR was obtained from Wako Pure Chemical Industries. Antibodies for phospho-AMPKα (Thr172) (40H9), total-AMPKα, phosphor-ACC (Ser79), total-ACC and LKB1 were obtained from Cell Signaling Technology. GAPDH antibody was obtained from Merck Millipore. Secondary antibodies for mouse IgG and rabbit IgG were obtained from Santa Cruz Biotechnoligy.

siRNA for DARP was obtained from Dharmacon, and the negative control siRNA (scramble) was obtained from Ambion.

### Cell culture

C2C12 mouse skeletal myoblast cell line was cultured in DMEM supplemented with 10% fetal bovine serum and 1% penicillin and streptomycin. Myogenesis was induced as described previously [10]. Briefly, when cells reached 70–80% confluency, the growth medium was switched to differentiation medium (DMEM supplemented with 2% horse serum and 1% penicillin and streptomycin) to induce myogenesis. Differentiation medium was changed every other day until cells well differentiate into myotubes (regularly for 5–9 days).

### Gene-silencing of DARP

Differentiated C2C12 myotubes (3–5 days after giving differentiation medium) were transfected with 50 nM siRNA for either DARP or scramble by using Lipofectamine RNAiMAX (Invitrogen), according to the manufacturer's instructions. The fresh differentiation medium was given after 24 h, and cells were incubated for another 48 h before use for experiments.

### Overexpression of DARP

Preparation of retrovirus was performed as reported previously [11]. Briefly, cDNA for targeting gene (DARP-FLAG) was subcloned into pMSCVneo vector (Clontech). GP2-293 packaging cells were transfected with DARP-pMSCVneo plasmid and pVSV-G plasmid (Clontech) using Lipofectamine 3000. In a parallel way, GP2-293 cells were transfected with GFP-pMSCVneo and pVSV-G plasmids to prepare viruses for negative control. Fresh growth medium was replaced 24 h after transfection, and cells were incubated for another 24h, followed by collection of the virus-containing culture medium. For infection, myoblasts of 50–60% confluency were incubated in the mixture of growth medium and the virus-containing culture medium at 1:1 ratio in the presence of 8 μg/ml polybrene for 24 h. Thereafter, cells were given fresh medium and incubated for 24–48 h followed by protein extraction.

### Animal study

All protocols for animal experiments were approved by the Ethics Review Committee for Animal Experimentation in Kyoto Prefectural University of Medicine. The total number of mice used in this study is 72. Detailed method for the generation of DARP-deficient mice was described previously [12]. Exon 1–3 of DARP gene were replaced by cDNA encoding LacZ and a pGK neo cassette in the targeted allele.

DARP-/- and WT mice (C57BL6) were fed with normal chow (containing 23.1% protein and 5.1% fat). For all the experiments using mice, mice were regularly anesthetized with isoflurane prior to every procedure to minimize suffering of the mice. When sacrifice the mice, mice were anesthetized with isoflurane and then sacrified by cervical dislocation.

### Treatment with AICAR *in vivo*

Treatment with AICAR *in vivo* was performed as described previously [13]. C57BL6 mice and DARP-/- mice were given an intraperitoneal injection of AICAR (0.25 mg/g) for 5 days prior to the ipGTT analysis. The condition of mice treated with AICAR was regularly monitored every day.

### Treatment with AICAR *in vitro*

Treatment with AICAR *in vitro* was performed as described previously [14]. Differentiated C2C12 myotubes transfected with scramble or DARP siRNA were treated with AICAR at a final concentration of 1 mM for 1 h, followed by protein extraction in RIPA buffer.

### Immunoblotting

Cell or tissue lysates were prepared in RIPA buffer containing protease and phosphatase inhibitors, and then immunoblotting was performed as described previously [15]. Lysates containing the same amount of proteins (~60 μg for culture cells and ~120 μg for quadriceps muscles) were subjected to SDS—PAGE, followed by transferring onto the nitrocellulose membrane. The membranes were blocked in 5% non-fat milk in TBS containing 0.05% tween20 at room temperature for 1 h. Membranes were then incubated with specific antibodies for target molecules. The dilution of primary antibodies was; phospho-AMPKα (1:1000), total-AMPKα (1:1000), phospho-ACC (1:1000), total-ACC (1:1000), LKB1 (1:1000), GAPDH (1:2000) and secondary antibodies for mouse IgG and rabbit IgG (1:4000).

### Quantitative RT-PCR

Quantification of mRNA expression of target genes was performed as described previously [16]. Total RNAs of skeletal muscle and C2C12 cells were isolated by using Trizol (Invitrogen), followed by purification with NucleoSpin RNA Clean-up (MACHEREY-NAGEL). Complementary DNA was synthesized from 0.5–1 μg total RNA using PrimeScript RT Reagent kit with gDNA Eraser (TaKaRa). PCR reactions were prepared by using KAPA SYBR FAST Master Mix Universal (KAPA BIOSYSTEMS) followed by the real-time PCR using Thermal Cycler Dice (TaKaRa). Nucleotide sequence of each primer is shown in Table 1. mRNA levels for target genes relative to 18S rRNA or actin was shown for all the experiments.

**Table 1:** Nucleotide sequences of primers.

GLUT1-forward	5’-CAATGGCGGCGGTCCTATAA-3’
GLUT1-reverse	5’-GAGAGACCAAAGCGTGGTGA-3’
GLUT4-forward	5’-ACTCTTGCCACACAGGCTCT-3’
GLUT4-reverse	5’-CCTTGCCCTGTCAGGTATGT-3’
LKB1-forward	5’-GGCATGGACACCTTCATCCA-3’
LKB1-reverse	5’-TCCTTCTTGACGTTGGCCTC-3’
DARP-forward	5’-AGCTGCTATAGAAGTACGGGATTTGC-3’
DARP-reverse	5’-GGACACTCGATAAGGTGCTCTAGGCA-3’
ANKRD2-forward	5’-CCCTGTGAATGAGGAGACATTCCTG-3’
ANKRD2-reverse	5’-CCAGAAGTTTCTCCAGTATCTCCATGTG-3’
Actin-forward	5’-AGCCATGTACGTAGCCATCC-3’
Actin-reverse	5’-CTCTCAGCTGTGGTGGTGAA-3’

### Metabolic measurement

The intraperitoneal glucose tolerance test (ipGTT) and insulin tolerance test (ITT) were performed as reported previously [17]. Briefly, for the ipGTT, mice were fasted for 8 h by depleting chows in the morning, and subsequently D-glucose was intraperitoneally administrated at the dose of 1.5 g/kg. Blood was collected at indicated time points, and blood glucose was measured by the glucose oxidase method (Sanwa Kagaku). For the ITT, mice in the absence of fasting treatment were subcutaneously given human insulin at the dose of 1 U/kg. Blood was collected at indicated time points, and blood glucose was measured. For quantitative analysis, the are-under-curve for ipGTT and ITT was also calculated.

### Measurement of glucose uptake in myotubes

Myotubes were serum-starved for 6 h and subsequently incubated with DMSO (control) or 2 mM AICAR for 15 min. Glucose uptake was determined by 2-deoxyglucose uptake with an enzymatic photometric assay by using 2-deoxyglucose uptake measurement kit (COSMO BIO Co. Ltd.) according to the manufacturer’s instructions.

### Measurement of (^14^C)-glucose oxidation in myotubes

Oxidation of glucose was assessed by measuring the production of ^14^CO_2_ from (^14^C)-D-glucose as described previously [18]. Briefly, myotubes transfected with either scramble or DARP siRNA were incubated with serum- and glucose-depleted medium containing fatty acid-free BSA and 5550 Bq/mL (^14^C)-D-glucose at a ratio of 1:2.5 in the presence or absence of AICAR (2 mM). To measure conversion of (^14^C)-D-glucose into ^14^CO_2_, a piece of Whatman papers wet with phenylethylamin/methanol (1:1) were taped onto the top of culture dish (35mm) to trap the CO_2_ produced during the incubation period. After incubation for 2 h, 200 μl of H_2_SO_4_ (4 mol/l) was added to release all the ^14^CO_2_, and cells were further incubated for 1 h. Subsequently, the seal was removed, and the pieces of Whatman papers were carefully collected and their radioactivities were measured by liquid scintillation counting.

### Statistical analysis

All data are presented as mean±S.E. Differences between groups were analyzed by the Student’s t-test or one-way ANOVA. P<0.05 was considered statistically significant.

## Results

### DARP is highly expressed in skeletal muscle and mature myotubes

**Fig 1:**
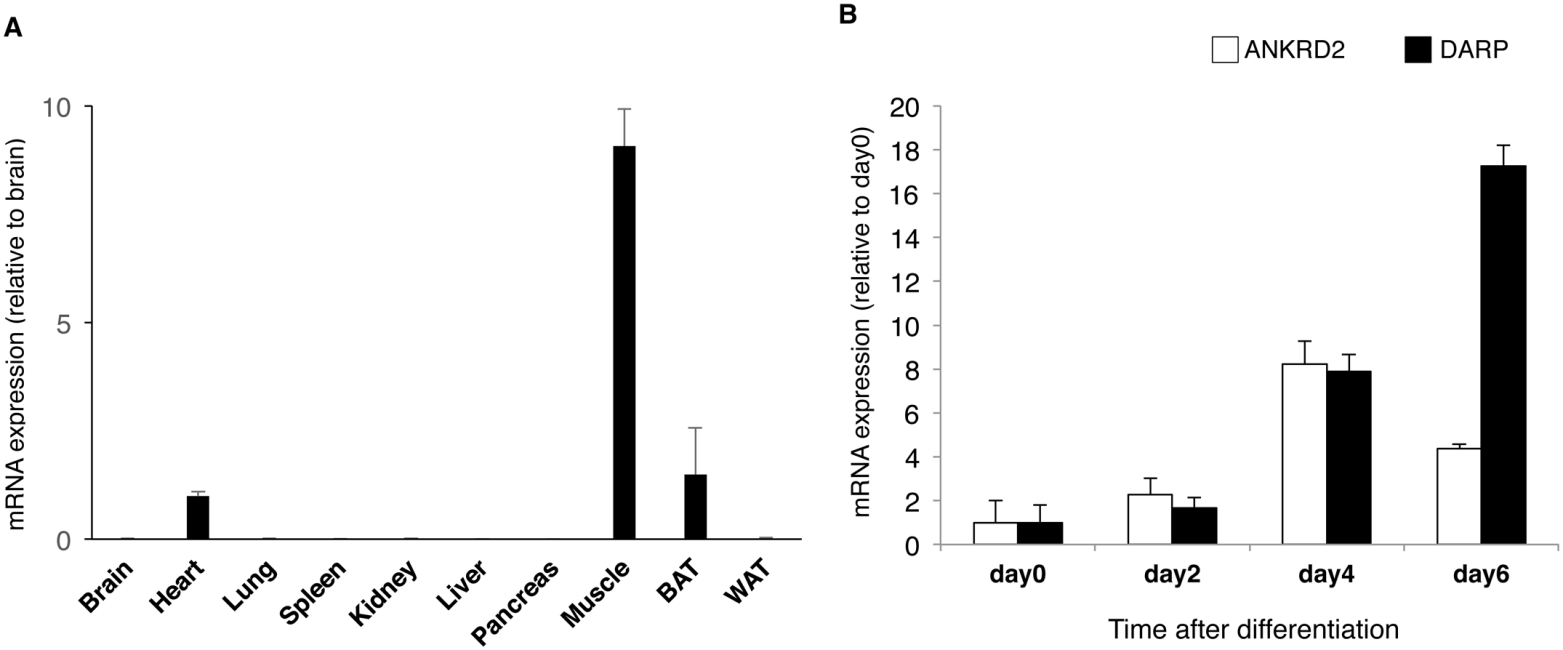
Expression of DARP in mouse tissues and myotubes. (A) Quantitative analysis for DARP mRNA expression in various mouse tissues (n = 3 each). DARP was predominantly expressed in skeletal muscle. (B) Quantitative analysis for ANKRD2 or DARP mRNA expression in C2C12 cells during myogenesis (n = 4 each). Expression of DARP was substantially enhanced during myogenesis, and was predominantly expressed in mature myotube. ANKRD2 showed the less degree of up-regulation during myogenesis comparing to that of DARP.

We previously reported that DARP is highly expressed in the skeletal muscle, and modest expression was detected in the heart and brown adipose tissue, assessed by northern blot analysis [7]. Therefore, we quantitatively analyzed the mRNA expression of DARP in mouse tissues, and found that DARP is expressed predominantly in skeletal muscle (Fig 1A). We then examined whether DARP expression is differentially regulated during myogenesis. C2C12 myoblasts expressed DARP at considerably low level, and DARP expression was substantially increased during myotube differentiation, indicating that DARP is predominantly expressed in mature myotube (Fig 1B). We also investigated ANKRD2 expression during myogenesis. ANKRD2 showed the lesser degree of upregulation during myogenesis, and undifferentiated myoblasts expressed ANKRD2 at a comparable level to mature myotubes (Fig 1B).

### Genetic inactivation of DARP improves glucose homeostasis in mice

**Fig 2:**
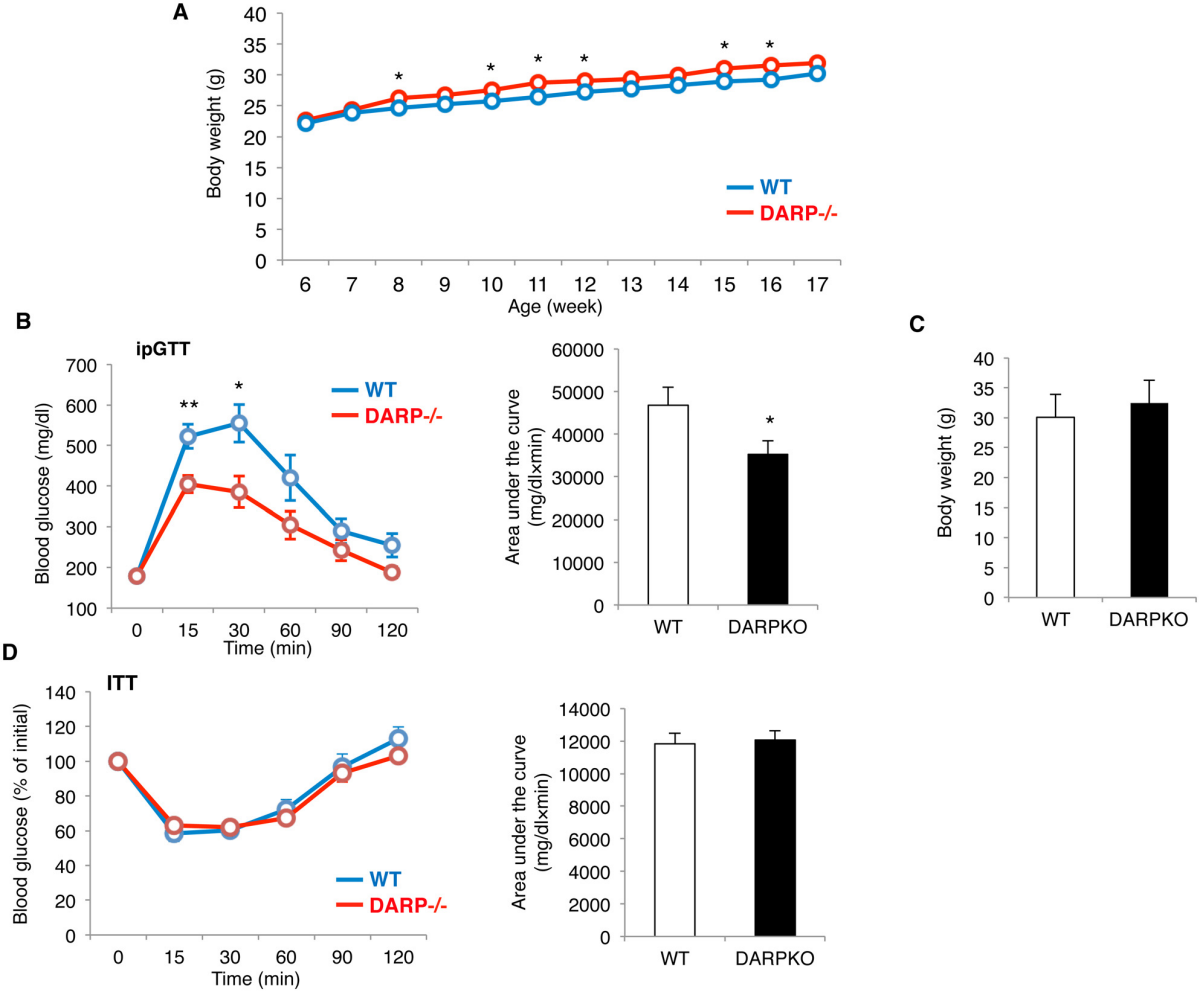
Inactivation of DARP improves glucose homeostasis. (A) Body weight of WT or DARP-/- mice at the indicated weeks of age. Body weight was similar between groups, while DARP-/- mice showed minimal and inconsistent increase in body weight relative to WT mice. *P<0.05 (n = 8 each). (B) Glucose tolerance test (ipGTT) was performed using WT or DARP-/- mice at the age of 24 week-old. *P<0.05, **P<0.01 (n = 6 for WT and n = 7 for DARP-/- mice). Quantification of the area-under-curve was also shown. DARP-/- mice showed better glucose tolerance comparing to that in WT mice. (C) Body weight of WT or DARP-/- mice at the time of ipGTT. There was no significant difference of body weight between WT and DARP-/- mice when ipGTT was performed. (D) Insulin tolerance test (ITT) was performed using WT or DARP-/- mice at the age of 20 week-old (n = 7 each). Quantification of the area-under-curve was also shown. Insulin sensitivity was similar in WT and DARP-/- mice.

To investigate a potential role of DARP in energy metabolism *in vivo*, we analyzed metabolic phenotypes of DARP-/- mice. DARP-/- mice showed body weight similar to wildtype (WT) mice, with a little tendency to increase (Fig 2A). We then performed glucose and insulin tolerance test using these mice. DARP-/- mice exhibited significantly reduced blood glucose levels after intraperitoneal administration of glucose comparing to those in WT mice, despite similar body weight (Fig 2B and 2C). However, insulin sensitivity assessed by insulin tolerance test was not different between DARP-/- and WT mice (Fig 2D). These results suggest that genetic loss of DARP enhances glucose tolerance without affecting insulin sensitivity.

### Loss of DARP activates AMPK in skeletal muscle

**Fig 3:**
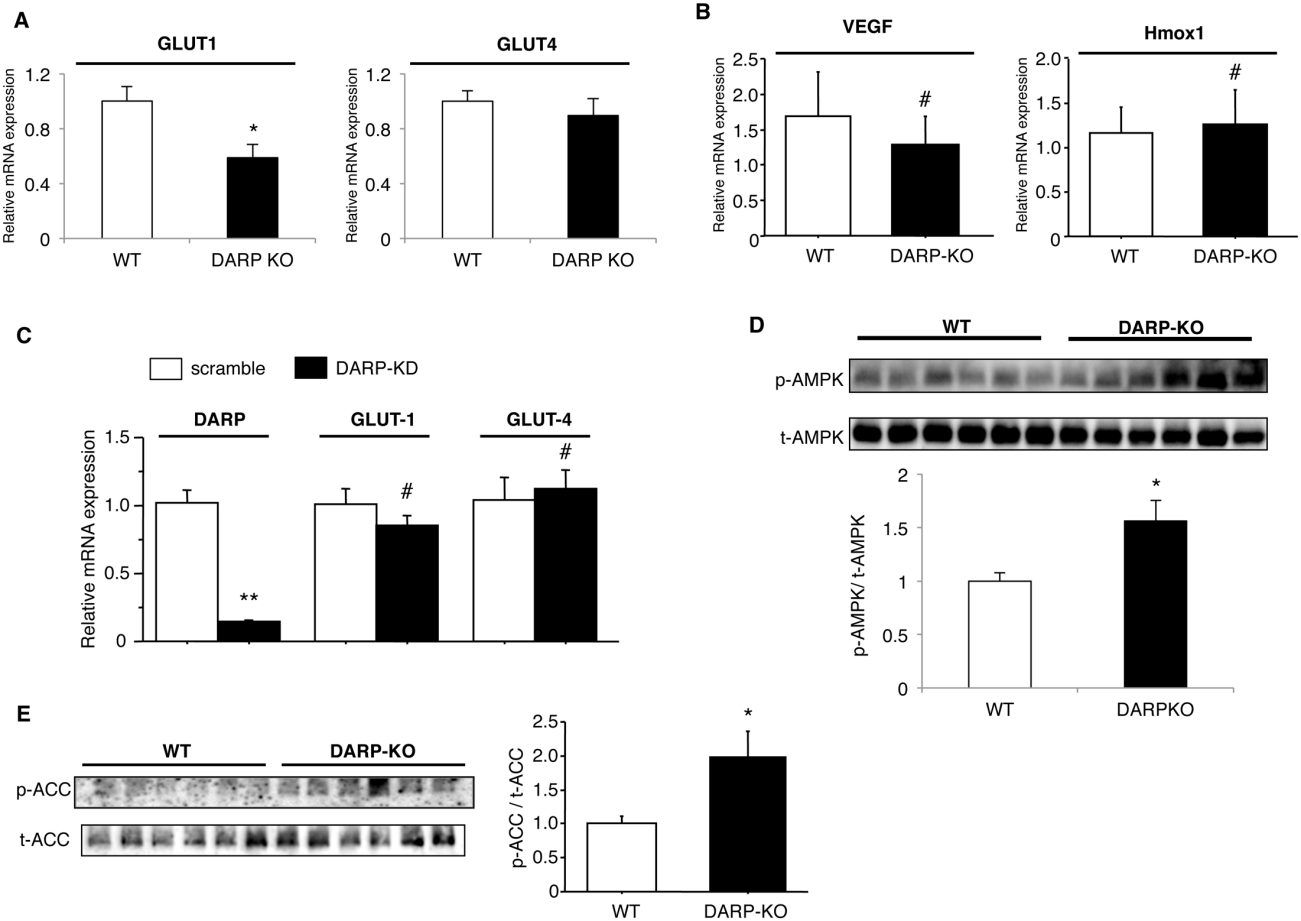
Loss of DARP leads to AMPK activation in skeletal muscle. (A) Quantitative analysis for GLUT1 and GLUT4 mRNA expression in skeletal muscle of WT or DARP-/- mice. *P<0.05 (n = 6 for WT mice, n = 5 for DARP-/- mice). Expression of GLUT4 in skeletal muscle of DARP-/- mice was similar, while GLUT1 expression in muscle of DARP-/- mice was significantly reduced comparing to those in WT mice. (B) Quantitative analysis for VEGF and Hmox1 mRNA expression in skeletal muscle of WT or DARP-/- mice. #Not significant (n = 5 each). (C) C2C12 myotubes were transfected with either negative (scramble) or DARP siRNA (DARP-KD), and then mRNA expression of DARP, GLUT1 and GLUT4 was quantitatively analyzed. **P<0.01, #Not significant (n = 4 each). (D) Phosphorylation of AMPK in skeletal muscle of WT or DARP-/- mice. *P<0.05 (n = 6 each). DARP-/- mice showed significantly accentuated AMPK phosphorylation in skeletal muscle comparing to WT mice. (E) Phosphorylation of ACC in skeletal muscle of WT or DARP-/- mice. *P<0.05 (n = 6 each). DARP-/- mice showed significantly accentuated ACC phosphorylation in skeletal muscle comparing to WT mice.

We then analyzed the expression of GLUT1 and GLUT4 in skeletal muscle. Expression of GLUT4 in skeletal muscle was not different between WT and DARP-/- mice, while GLUT1 expression was reduced in DARP-/- mice comparing to that in WT mice (Fig 3A). Because GLUT-1 expression is regulated by HIF-1α, we examined the expression levels of other HIF-1α target genes in skeletal muscle of these mice. The expression of vascular endothelial growth factor (VEGF) and heme oxygenase-1 (Hmox1) in skeletal muscle was similar in WT and DARP-/- mice (Fig 3B). We also analyzed expression of GLUT1 and GLUT4 in C2C12 myotubes transfected with either scramble or DARP siRNA. Gene-silencing of DARP did not affect the expression of both GLUT1 and GLUT-4 in C2C12 myotubes (Fig 3C). These data suggest that there is no direct link between DARP and HIF-1α/GLUT1. Of note, DARP-/- mice demonstrated significantly accentuated phosphorylation of AMPK in skeletal muscle comparing to that in WT mice (Fig 3D). In consistent with the enhanced AMPK activity, phosphorylation of acetyl-CoA carboxylase (ACC) was also accentuated in skeletal muscle of DARP-/- mice (Fig 3E).

**Fig 4:**
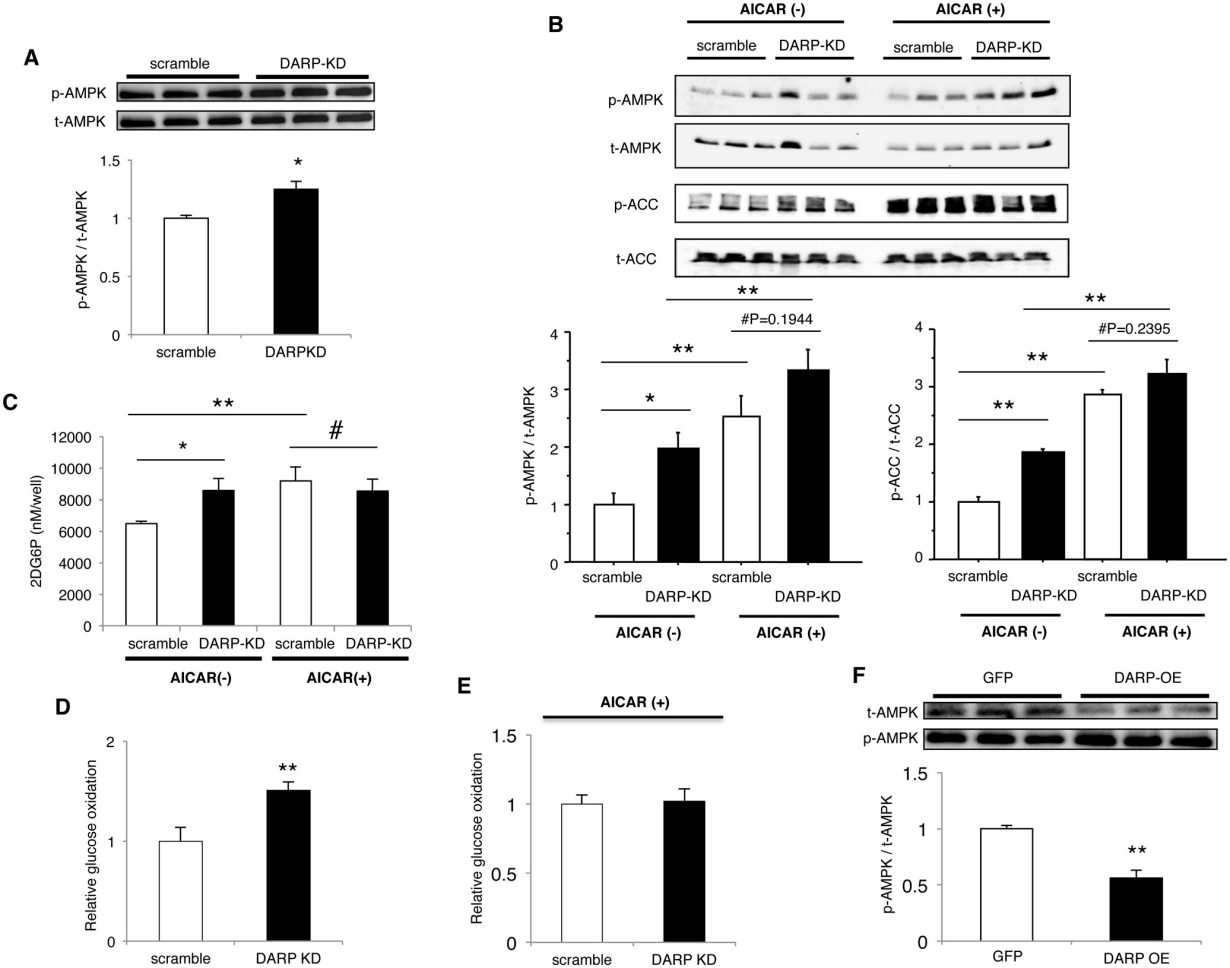
DARP regulates AMPK activity in C2C12 cells. (A) Phosphorylation of AMPK in C2C12 myotubes transfected with either negative (scramble) or DARP siRNA (DARP-KD) was assessed by immunoblotting. Silencing of DARP significantly enhanced the phosphorylation of AMPK. *P<0.05 (n = 3 each). (B) Phosphorylation of AMPK and ACC in C2C12 myotubes in the presence or absence of AICAR was assessed by immunoblotting. Silencing of DARP significantly enhanced the phosphorylation of AMPK and ACC in the absence of AICAR. Treatment with AICAR enhanced phosphorylation of AMPK and ACC in C2C12 myotubes, and the difference of their phosphorylation between scramble and DARP-KD cells disappeared when treated with AICAR. *P<0.05, **P<0.01, #Not significant (n = 3 each). (C) Glucose uptake in C2C12 myotubes was assessed by measuring 2-deoxyglucose uptake using an enzymatic photometric assay. Cells were transfected with either scramble or DARP siRNA (DARP-KD), and the glucose uptake was analyzed in the presence or absence of AICAR. Silencing of DARP significantly increased glucose uptake in C2C12 myotubes comparing to control cells. *P<0.05 (n = 5 each). Treatment with AICAR increased glucose uptake in control cells and abolished the increased glucose uptake induced by DARP-silencing. **P<0.01, #Not significant (n = 5 each). (D) Glucose oxidation in C2C12 myotubes was assessed by ^14^CO_2_ production from ^14^C-D-glucose. Cells were transfected with either scramble or DARP siRNA (DARP KD). DARP-silencing enhanced the glucose oxidation in C2C12 myotubes. **P<0.01 (n = 5 for scramble, n = 4 for DARP KD). (E) Treatment with AICAR abrogated the increase in glucose oxidation induced by DARP-silencing (n = 3 each). (F) C2C12 myoblasts were infected with retroviruses carrying GFP (GFP) or DARP (DARP OE) gene, and then phosphorylation of AMPK was assessed by immunoblotting. Overexpression of DARP significantly reduced the phosphorylation of AMPK. **P<0.01 (n = 3 each).

Because AMPK mediates insulin-independent glucose uptake in muscle [19–21], these results suggest that loss of DARP might enhance glucose tolerance by activating AMPK. Therefore, we further examined the role of DARP in AMPK activation. Gene-silencing of DARP significantly enhanced phosphorylation of AMPK and ACC in C2C12 myotube (Fig 4A and 4B). Furthermore, we found that DARP-silencing significantly increased glucose uptake in C2C12 myotubes (Fig 4C). Treatment with AICAR, an AMPK activator [20], enhanced phosphorylation of AMPK and ACC in C2C12 myotubes, and the difference of their phosphorylation between control and DARP-knocked down cells disappeared after treatment with AICAR (Fig 4B). Accordingly, the increase of glucose uptake by DARP-silencing was abrogated by the treatment with AICAR (Fig 4C). In addition, glucose oxidation was enhanced by DARP-silencing in C2C12 myotubes, and this increase of glucose oxidation was also abolished by AICAR-treatment (Fig 4D and 4E). In contrast, overexpression of DARP significantly reduced the phosphorylation of AMPK in C2C12 myoblasts comparing to the control GFP-transfected cells (Fig 4F). These data strongly suggest that DARP negatively regulates AMPK activity in the skeletal muscle.

### Enhanced AMPK activity is attributable to the better glucose homeostasis in DARP-/- mice

**Fig 5:**
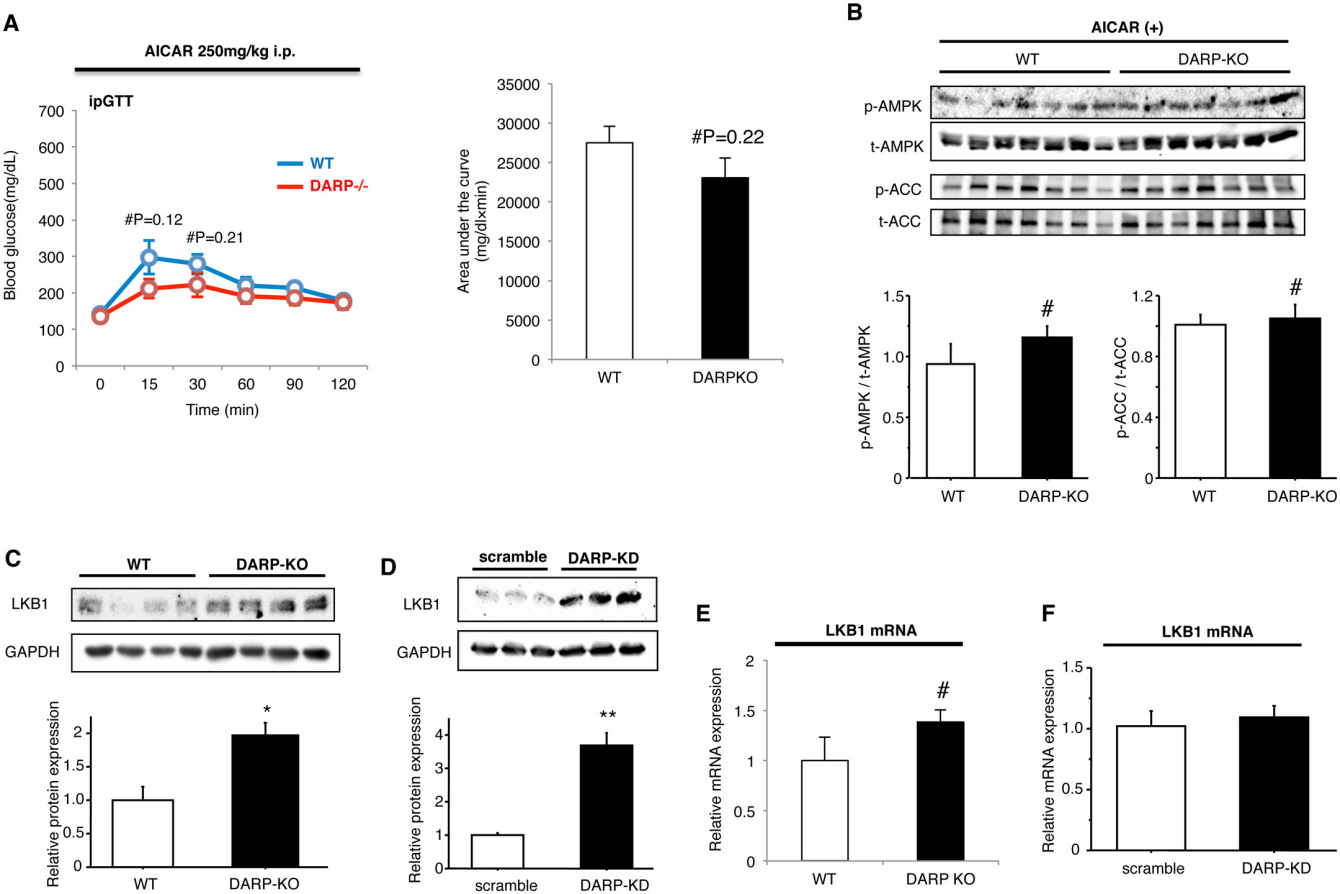
Enhanced AMPK activity is attributable to the better glucose homeostasis in DARP-/- mice. (A) Glucose tolerance was analyzed in WT or DARP-/- mice treated with AICAR at the age of 24 weeks old (n = 6 for WT mice, n = 7 for DARP-/- mice). Administration of AICAR abolished the better glucose tolerance in DARP-/- mice. (B) Phosphorylation of AMPK and ACC in skeletal muscle of WT or DARP-/- mice treated with AICAR was assessed by immunoblotting. #Not significant (n = 7 each). (C) LKB1 expression in skeletal muscle was assessed by immunoblotting. Protein expression of LKB1 increased in skeletal muscle of DARP-/- mice comparing to that in WT mice. *P<0.05 (n = 4 each). (D) LKB1 expression in C2C12 myotubes transfected with either negative (scramble) or DARP siRNA (DARP-KD) was assessed by immunoblotting. Protein expression of LKB1 increased in DARP-KD myotubes comparing to that in scramble control cells. **P<0.01 (n = 3 each). (E) Quantitative analysis for LKB1 mRNA expression in skeletal muscle of WT or DARP-/- mice (n = 6 for WT and n = 5 for DARP-/-). LKB1 expression in skeletal muscle was not significantly different between WT and DARP-/- mice. (F) Quantitative analysis for LKB1 mRNA expression in C2C12 myotubes transfected with either negative (scramble) or DARP siRNA (DARP-KD). LKB1 expression in myotubes was not significantly different between scramble and DARP-KD cells (n = 4 each).

We finally investigated whether AMPK activation is attributable to the enhanced glucose tolerance observed in DARP/- mice. Administration of AICAR abolished the better glucose tolerance in DARP-/- mice, indicating that enhanced AMPK activity largely accounts for the enhanced glucose tolerance induced by DARP-deletion (Fig 5A). We confirmed that phosphorylation of AMPK and ACC in skeletal muscle was not different between WT and DARP-/- mice after the treatment with AICAR (Fig 5B). To investigate the mechanisms responsible for the modification of AMPK activity by DARP, we examined the expression of LKB1, a major upstream kinase that phosphorylates AMPK [4,22]. Gene-silencing of DARP significantly increased protein expression of LKB1 in skeletal muscle *in vivo* and C2C12 myotubes *in vitro*, suggesting that DARP modifies AMPK activity at least partially by modulating LKB1 expression in skeletal muscle (Fig 5C and 5D). In contrast, mRNA expression of LKB1 was not affected by DARP-silencing, indicating that DARP modifies the LKB1 expression at protein levels but not mRNA levels (Fig 5E and 5F).

Taken together, we unveiled a previously unknown function of DARP in the regulation of glucose homeostasis by modulating AMPK activity, shedding light on DARP as a new therapeutic target for the treatment of blood glucose dysregulation in diabetic patients.

## Discussion

In this study, we identified that DARP regulates glucose disposal in skeletal muscle and thus modifies the glucose homeostasis by modulating AMPK activity. Because reduced glucose disposal in skeletal muscle largely accounts for the glucose intolerance in diabetic patients [2,23], DARP could be a novel pharmacotherapeutic target for the treatment of diabetes mellitus.

By using the PCR-based subtractive hybridization, we previously identified DARP as a nuclear protein whose expression was differentially regulated in obese and insulin resistant animals [7]. DARP expression was enhanced in skeletal muscle of obese rat, and reduced AMPK activity in skeletal muscle has been reported in obese and diabetic patients as well as in mice with metabolic syndromes; therefore, DARP may play a causal role in the dysregulation of AMPK under obese and/or diabetic conditions. Also, DARP expression was reduced in skeletal muscle by fasting, while it increased beyond the basal level after re-feeding in lean mice. AMPK plays a vital role in the metabolic adaptation to changes in nutritional status [20,24]. The AMPK system is regulated by AMP-to-ATP ratio through allosteric and non-allosteric phosphorylation of a key threonine residue, and thus functions as a master energy sensor governing the adaptive metabolic changes to fasting [24,25]. Fasting activates AMPK, which inhibits anabolic pathways and enhances catabolic processes, to restore ATP levels [24]. Because DARP likely inhibits AMPK activity, fasting-induced decline in DARP expression may contribute to the efficient activation of AMPK system during fasting. Also, rebound increase of DARP expression might be helpful to sufficiently inactivate AMPK system after re-feeding. Therefore, DARP might be involved in the metabolic adaptive responses to the changes in nutritional status as well through the fine-tuning of AMPK activity in skeletal muscle.

There are two other members in MARP family; CARP and ANKRD2 [8,9]. Despite the striking expression pattern in the heart and skeletal muscle, and possible association with diseases such as cardiac hypertrophy, dilated cardiomyopathy, skeletal muscle myopathy and muscular dystrophy, physiological roles of CARP and ANKRD2 remain unclear [12]. CARP was initially identified as a cytokine-inducible gene in endothelial cells, and later revealed to be a downstream of the homeobox gene Nkx2.5 and regulate the expression of cardiac genes [12,26]. ANKRD2 has been identified as a mechanical stretch-responsible gene in skeletal muscle, and its expression was differentially regulated in response to exercise, eccentric contraction and denervation [27]. Recently, it has been reported that mice with triple knockout for CARP, ANKRD2 and DARP are viable and fertile, and they showed no macroscopic as well as microscopic abnormalities in the heart and skeletal muscle [9,12]. Detailed analysis revealed that loss of all MARP family members caused a greater degree of torque loss after a bout of eccentric contraction in muscles, but no significant difference in cardiac function and hypertrophy was detected in these mice [9,12]. Studies using gene knockout mice are needed to elucidate whether CARP and ANKRD2 are also involved in the glucose homeostasis and/or energy metabolism in the heart and skeletal muscle.

In summary, we have identified that DARP modifies glucose homeostasis by modulating AMPK activity in skeletal muscle. Also, DARP may play a role in adaptive metabolic changes to nutritional variation through a fine-tuning of AMPK activity. However, detailed molecular mechanisms underlying the DARP-mediated modification of AMPK activity remain to be elucidated although LKB1 is likely involved in these mechanisms. Also, we used DARP-null mice in this study, but muscle-specific knockout of DARP is needed to clearly show the DARP function in skeletal muscle. Further analyses, especially for the pathway in AMPK phosphorylation and dephosphorylation are required to identify molecular mechanisms how DARP modifies AMPK activity and systemic glucose homeostasis.
